# What Happened to International Labour Standards and Human Rights at Work?

**DOI:** 10.1007/978-3-030-55400-2_3

**Published:** 2020-10-17

**Authors:** Kari Tapiola

**Affiliations:** 1 Office of the President of the Republic of Finland, Helsinki, Finland; 2 Faculty of Law, University of Helsinki, Helsinki, Finland; International Labour Organization, Geneva, Switzerland

## Abstract

The social rules of a universal market economy, created by globalization, are based on the standards adopted by the ILO since 1919. Among them a special role belongs to fundamental principles and rights at work, comprised in an ILO Declaration in 1998. They provide for freedom of association, collective bargaining and the elimination of child and forced labour and discrimination. There is a growing debate on how other standards should be linked to fundamental rights and not seen as less important instruments. Technical cooperation has demonstrated that, in any case, implementing fundamental rights leads to strengthening of law and practice on wages, social security and occupational safety and health. All international labour standards (Conventions, Recommendations and Protocols) are derived from the labour principles of the ILO Constitution, and they are closely connected with one another. While the role of the state remains crucial—especially in times of crisis—much of the implementation of labour standards should be achieved through collective bargaining and other negotiations, while voluntary agreements between the social partners are generally legally binding.

## Introduction

There has been debate on the international labour standards of the ILO for the whole hundred years of its existence. This has concerned the choice of topics themselves, the detail in which the norms have been set out, and their ratification and subsequent application and supervision. During the last 40 years the debate has been moving from one extreme to another. The post-World War II consensus on the need to maintain a balance between economic and social progress was an element in reconstruction and of industrialized countries’ growth and welfare. As of the 1980s, this consensus broke down. With increased emphasis on market forces, labour standards started being seen as rigidities and impediments to growth. Once the Cold War ended, a question in the early 1990s was posed in almost brutal terms: now that the common enemy—communist state power—was gone, was there still a need for the ILO and its standards?

Socialist political and economic structures crumbled as of 1989 at a pace no one had foreseen. This in turn brought about a new transparency and a new openness of the entire world economy, enabled by technological change and the utilization of real-time sourcing and production. However, the good news of the spread of democracy and markets was accompanied by a shock when especially Western consumers found out that new and affordable products from emerging countries were—or could be—produced by very young children in miserable conditions. Consumers reacted for moral reasons; workers reacted because cheap imports affected their jobs. All of a sudden there was a call for universal rules on labour again. After having first been sidelined in the frenzy of transition, fundamental rights at work became a hotly contested issue in globalization and international trade.

As the world trading system was being reorganized, the question of fundamental rights at work turned around the call for a “social clause”, which was designed to make access to world trade conditional on observance of labour rights. The world trade lobby succeeded in keeping the issue away from the World Trade Organization, which was set up in 1995. Efforts to keep the issue alive were largely confined to the ILO, which remained, as before, the custodian of international labour standards.

The contents of fundamental human rights at work were clarified by the ILO in the Declaration on Fundamental Principles and Rights at Work, adopted in 1998.^1^ The follow-up to the Declaration boosted these standards by promising technical cooperation, with which they now became increasingly linked. The ILO introduced an extensive programme of assistance to developing countries for freedom of association, the right to collective bargaining, freedom from forced and child labour and multiform action against all forms of discrimination at work.

These fundamental rights—or “core labour standards” as they were also called—were universally accepted as an element of the new globalized world order. Support for applying them and even pressure to do so was assisted by extra-budgetary resources from the industrialized countries. For many of them, this was the second best option once trade sanctions were discouraged. Fundamental rights made their way into international documents governing trade, investment and cooperation. They were seen to translate the social dimensions of the new world economy, while promoting them was akin to the role of the ILO after the two World Wars.

To what extent this new focus on social justice was genuine or simply words, or opportunism, is anybody’s guess. Yet the formula of ILO technical cooperation for a level playing field for trade and a means of raising standards among the emerging participants of the trading system was appealing. Soon over half of the technical cooperation of the ILO was in the field of fundamental rights at work, most of it aimed at eliminating child labour.^2^

Since the financial crisis, which burst out in 2008, and since the recent worldwide political slide towards extremism, nationalism, xenophobia and brutal egoism, we have again heard less about rights at work.

However, the spell of neo-brutalism that we are living through springs from the same source as the desire for social and labour rules. Both are driven by fear that the forces of globalization have become an existential threat to individuals and societies. These concerns arise from uncertainties about employment, incomes and maintenance of social status in an increasingly volatile world. Two images illustrate what has happened. Products from subcontractors with workers in shabby conditions in underdeveloped societies have been flooding the markets. Especially since 2015, a highly visible flow of migrants and refugees across borders has also been occurring.

These images have been destabilizing industrialized countries while at the same time allowing a glimpse of the hopes and despairs of the developing world. Both workers and entrepreneurs in all countries have been affected. Most vulnerable have been the categories accounting for a significant amount of employment: the self-employed and micro- and small and medium enterprises. At the same time, while the line dividing opportunity and exclusion has remained endemic in less fortunate countries, it has also cut through the richest societies of the world.

The question of fundamental principles and rights at work is an issue for each and every society, especially taking into account modern slavery and trafficking, zero hours employment contracts, “Uberization” of urban services, the platform and gig economies and profiling due to political suspicions, as well as harassment and violence at work. Our new divisions are between the wealthy and fortunate on the one hand and the struggling and excluded on the other hand at all points of the compass. One dividing factor remains the way and extent to which labour standards are applied. In this complex situation the linkages between standards designed as fundamental and other—more “technical”—standards are even more topical than in earlier times. In a nutshell, these are so interlinked that it is not possible to have any one without the others—as I shall aim to demonstrate in this chapter.

## Development of International Labour Standards

International labour law was a novel notion in 1919. The standards adopted by the ILO are derived from the labour principles of its Constitution.^3^ These principles had in turn been formulated by the trade unions during the First World War and were originally proposed as the “labour clauses” of the Peace Treaty. The Versailles Treaty did not produce a lasting peace or universal happiness, but at least the system of rights expressed in international labour standards was born.

The labour principles of 1919 are the basis of the International Labour Code. These principles cover a broad scope of labour rights, starting with freedom of association, hours of work, employment policy, maternity protection, labour inspection, social security provisions, minimum age for employment and the health and safety of different categories of workers. Most of the labour legislation and practice in the world today has been shaped by these principles.

For many decades the corpus of international labour standards grew at a regular pace. Most issues were treated by either a Convention or a Recommendation, or in a few cases a Protocol. Conventions become binding through national ratification; Recommendations form an integral part of the standards system and although they are not binding, in principle they should apply to everyone. Other instruments, such as Declarations and codes of practice, have complemented the ILO toolkit. They do not have the status of labour standards, but they do give guidance for treating labour and social issues at national level, including by labour legislation. From the outset, this system has been a combination of what we occasionally call “hard” and “soft” law.

## Human Rights Standards

After the Second World War, the normative foundation of the ILO was aligned with the need to reaffirm democratic rights and promote development. What we know as fundamental rights at work were created by standard-setting on human rights after the 1944 International Labour Conference in Philadelphia. This coincided with the early years of the United Nations, democratization, decolonization and the Cold War.

Discrimination and forced labour had come into a new focus during the labour and extermination camps of the Second Word War. As decolonization proceeded, the heavy weight of discrimination and of imperial-age economic interests continued to hamper the achievement of true national sovereignty.

Decolonization created the African group in the United Nations and the ILO. When racial segregation not only continued but became increasingly brutal in South and Southern Africa, the issue of discrimination rose to the top of the agenda. South Africa was forced to withdraw from the ILO in 1964, but apartheid remained prominently on the agenda through special procedures in which trade unions and employers participated. At the same time, the United States had to accept significant measures for desegregation in its Southern States.

The shattering war-time experiences of racial discrimination, forced labour and denial of rights of not only workers but also employers played an important role in building a consensus on human rights at work. Employers had still been ambivalent about the role of Mussolini’s fascist corporations in Italy, but subsequently the course of the war showed that their organizations could be equally threatened by totalitarian régimes. In 1919, the language of the labour principles of the Constitution had affirmed that freedom of association was a right of workers and employers alike.^4^ In the early Cold War years, the ILO recognized that the independence of employers was also covered by the concept of trade union rights.^5^

The questions of freedom of association and forced labour found new relevance because of the practice of communist countries. What had been a reaction to the atrocities of the fascist regimes carried over to discussion on social, individual and economic liberties in reconstruction and economic development. In the labour field, during the Cold War, certain human rights—above all freedom of association—defined the side on which you were between the market economy and communism. This placed the main part of the trade unions on the same side of the divide as employers. In practice this determined much of the standard-setting after 1948 well beyond the fundamental principles and rights at work. Multiple compromises were called for in the period between 1945 and 1989—between employers and workers, between industrialized and developing countries, and between radical and moderate elements of—especially—the Workers’ Group.

During the long tenure of David Morse as Director-General (1948–1970), the ILO stressed employment, skills and social policies which were guided by the tripartite engagement at the national level of governments, employers and trade unions.^6^ Significant normative work was done on labour inspection and administration, social security, occupational safety and health, employment and labour market policies, gender equality, paid educational leave and holidays with pay.

These were the building blocks of a liberal social development model, based on a negotiated balance of interests between and within different constituent groups of the ILO. Fifty years ago it earned the ILO the Nobel Peace Prize.

Soon thereafter, however, global changes and technological innovation conspired to change the parameters. Multinational enterprises moved production across national borders and, in extreme cases, could cause political upheavals, such as the coup d’état against Salvador Allende in Chile in 1973. Oil crises and debt crises started shaking expectations of stability and continuous growth and prosperity.

The result was a relatively rapid sea-change. One of the reasons for this was the realization that in large parts of the world trying to reproduce the industrialized countries’ development model did not lead to sustained employment. In the 1980s, with—especially—European growth lagging, calls for giving more freedom to market forces were accompanied by new technology, which allowed real-time cross-border production and severed many physical employer-employee links. This was followed by accelerated liberalization of trade and capital movements.

## Establishing the Social Dimension

In the new situation after the end of the Cold War and the global opening of markets, the Declaration on Fundamental Principles and Rights at Work of 1998 and its follow-up activities expressed an underlying aim of the ILO: to strengthen the social dimension of international economic and social policies. After the First World War, the founding of the ILO had brought a social dimension to peacemaking. After the Second World War, the ILO had provided the social dimension of both reconstruction and—once decolonization got under way—of development. The Cold War ended without a political or social peace deal with “labour clauses”. The notion of universal fundamental rights at work had to assume much of the role of providing for a social dimension. In the debate on standards versus deregulation in the global market, it worked as a smart battle ram, piercing resistance to all kinds of standards. But it could not serve as a broad social contract.

The 1998 Declaration contained consensus on a short-list of “fundamental” social and labour rights: freedom of association, the right to collective bargaining, abolition of forced labour, elimination of child labour and rejection of discrimination in employment and occupation, including equal pay for work of equal value between women and men.

Each of these four categories of rights was linked to a Convention. Three of the four categories had Conventions which at least in principle were universally acceptable: Nos 87/98 on freedom of association and collective bargaining, Nos 29/105 on forced labour and Nos 100/111 on discrimination and equal remuneration. Child labour was attached to this short list a bit later.

The years when David Morse, Wilfred Jenks and Francis Blanchard led the ILO saw discussion on trade and labour standards. The debate did not in any way start only in the 1990s. Additionally, both recognized that besides human rights at work such issues as a living wage, labour inspection, health and safety and basic social security should be addressed. These were all derived from the original Constitution of the ILO but the Conventions on them did not enjoy the same status and consensus as the human rights Conventions.

In the early 1990s it was politically necessary to have a manageable list of standards because of the de facto link to international trade. The OECD produced a study which showed how difficult it could be to deal with an extended list of up to ten categories of standards, in particular if the link to trade would mean intensified enforcement measures through existing or new supervisory mechanisms of Conventions.^7^

Conventions on wages, social security or occupational safety and health were not as widely ratified, and even the leading ones covered only parts of the strategic decent work objectives to which they belonged. A large number of Conventions, which all had several technical provisions, could thus have become new arguments in trade policies. As the stated aim of the post-Cold War debate was liberalization of international trade, new entrants to the markets feared that raising too many social issues could negate their advantages. The four “fundamentals” were known and reasonably safe. A linkage with social security provisions, occupational safety and health or the need to ensure a living wage would have been one bridge too far.

Making an operative link to trade through any kind of sanctions mechanism was anathema both for emerging countries and employers. However, promoting the ratification and application of fundamental Conventions was not. The four categories had been established at the United Nations Social Summit in Copenhagen in 1995, with heavy involvement by ILO constituents. The Conventions on freedom of association, collective bargaining, child and forced labour and discrimination were formally recognized as human rights. All countries which had ratified them should fully respect their legal obligations while all others should do their best to live up to their principles.^8^

After Copenhagen, ILO Director-General Michel Hansenne launched a ratification campaign on the relevant Conventions. At that moment the Minimum Age Convention No. 138 was quietly added to the list of “core” Conventions as the then applicable standard on child labour.

At the same time, a need clearly existed for an additional human rights standard on child labour. The earliest labour legislation at the beginning of the nineteenth Century had been on child labour. In the ILO, child labour had always been treated through Conventions on the minimum age for employment. This reflected the fact that the age of moving into full-time employment should permit compulsory education, particularly after universal schooling had become the norm towards the end of the nineteenth Century. Sectoral Conventions adopted by the ILO were in 1973 taken over by the Minimum Age Convention No. 138. However, the ratification rate of this Convention was meagre, and it had not been recognized as a priority Convention subject to more intense scrutiny.

When the need for benchmarks to define what was most intolerable in the work of children came up, the Minimum Age Convention was found to contain a useable formula establishing age limits as well as flexibility linked to levels of national development and the nature of work. When in 1999 a new Convention on the Worst Forms of Child Labour No. 182 was adopted, it built upon this Convention instead of revising it. The ratification rate of the Worst Forms of Child Labour Convention has broken all records, also delivering ratifications of the Minimum Age Convention at a rate which had been unthinkable before.

Child labour was included in the four categories of fundamental rights because of the emotionally strong effect of finding out that child labour was a factor in trade. It was possible to argue that while it was a normative issue, its eradication called for time-bound technical assistance programmes. It was also the item which had the lowest level of political resistance: no régime was really threatened by its elimination. Even the Gulf States agreed to remove young children from camel jockeying in widely popular competitions, sometimes replacing them with robots.

The 1998 Declaration was also supposed to offer an easier way than ratification for countries to express their commitment. Countries could report annually to the Governing Body on their efforts to realize the principles of the Conventions, and they could signal the kind of technical cooperation they felt would be necessary to support these efforts. This innovative method could be seen as positive encouragement instead of reporting on ratified Conventions, which tended to be more investigative and often controversial.

Sometimes what happens is the unexpected. The new innovative and—presumably customer-friendly—reporting mechanism was in practice soon overtaken by what could be called an escape into the safety of the established mechanism. In short, instead of availing themselves of the new and apparently “softer” opportunity, a large number of countries preferred to ratify the fundamental Conventions instead. This was a case of preferring the devil you know to the devil you don’t. As a result, the overall ratification rates of the eight fundamental Conventions shot up to over 90%, with child and forced labour reaching nearly universal levels.

## The Effects of Technical Cooperation

Such a change in the attitude to ratifications would not have been possible without increased linkage to technical cooperation. The traditional view had been that ratification of a Convention would take place only after national law and practice had been made to conform to its requirements. When technical cooperation became a recognized part of this process, and was increasingly recommended by the standards supervisory mechanism, it became acceptable to ratify at an earlier stage once the political will was there. Technical cooperation would then ensure implementation. Financing was available, as the industrialized donor countries responded rather generously to what they saw as a positive link created between trade and labour standards.

What actually happened was that in many cases, instead of countries sliding into a conflict and economic and trade sanctions, deficiencies in labour standards led to agreements on technical cooperation; this in turn provided a compelling argument against sanctions. The credibility of such arrangements was seen to be guaranteed by the fact that reporting to, and discussion by, the standards supervisory bodies of the ILO would soon reveal how sincere the engagement of the country concerned was.

Since the early 2000s, fundamental principles and rights at work have become a part of a global *aquis*. Today it is almost unthinkable that, for instance, any politician or scholar could defend child labour by referring to cultural differences or even economic necessity. Likewise, a world-wide consensus has developed on the need to take action against human trafficking, which is one of the modern-day features of forced labour.

Despite the misgivings of the emerging world, labour standards as seen through the prism of the 1998 Declaration have found their way to trade legislation as well. This has not taken place so much through outright conditionality as through trade preferences accorded to countries which desire to respect fundamental labour standards. The European trade systems opted to use the ILO’s short-list for trade preferences. Most bilateral or regional free trade treaties have followed the same pattern, either referring to the fundamental Conventions or the 1998 Declaration. The United States, however, has continued to apply a broader set of criteria than the fundamentals to its trade policies. This included labour inspection. When the US trade unions filed a trade petition because an ultraliberal 2006 Georgian Labour Code—later amended—violated freedom of association principles, the case was mainly about labour inspection which the Georgian reformers of the former communist system had scrapped.^9^

What I am arguing here is that the focus on fundamental rights at work safeguarded the role of international labour standards in general. The standards and their application were seen as a remedy to the problems of globalization and not as a complication or rigidity. In addition, the wave of ratifications of the fundamental Conventions led many countries to review their position on ILO Conventions in general. The factual blockage on new ratifications of all Conventions, which characterized especially the 1980s and 1990s, is no longer there.

## Beyond Fundamental Rights

If we assume that, by now, the case has again been made for standards, it should also be possible to expand the scope beyond fundamental rights. This does not mean going away from them, but rather beyond them. Actually, this needs to be done even for the sake of the fundamentals themselves. Their implementation needs to reach out to the full scale of decent work. Whether in the informal or the formal economy, anywhere we want to go with the fundamentals, we quickly arrive at the whole normative basis of society.

We are facing a situation which may at first appear to be contradictory. Frustration with detailed regulation, and the obligations of ratifying states compared with a large number of non-ratifying competitors, had led to a desire to simplify the basic rules of the game around a short-list of fundamental standards. This was by and large successful. It saved the credibility of the standards system of the ILO, not least due to assistance for countries which chose to admit that the problems they experienced in applying standards were due to insufficient capacity and not inadequate political will.

However, assistance also showed that when you start to implement fundamental rights, you are faced with a large number of practical situations and have to venture into a field which is covered with other regulations and practices. To give a simple example: after 1998 some millions of US dollars were made available for freedom of association in Indonesia. One key tool of the ILO is the Committee on Freedom of Association of the Governing Body, but you do not spend millions on advising how to write complaints to the CFA. The outcome was a broad programme of social dialogue, which included support for the development of trade unions and employers’ organizations.

## A Linkage to Adam Smith

It is instructive to note that both the case for workers’ right to organize and linkages between fundamental and other rights were outlined in 1776 by Adam Smith in his *The Wealth of Nations*. Smith was the prophet of liberal economics and free trade to the extent that British Prime Minister Margaret Thatcher reportedly carried his book in her voluminous handbag. Writing about the wages of labour, Adam Smith observed that workers wanted to gain as much as possible in wages while employers conceded as little as possible. Both had an interest in combining (organizing) but Parliament had prohibited the workers from doing so. The masters in turn could always collude—and tacitly did so—to keep the price of labour down.^10^

If workers were bereft of their wages, their existence could become untenable in a matter of days while employers could weather long periods of conflict. Workers could act with the “folly and extravagance of desperate men who must either starve or frighten their masters into an immediate compliance with their demands”.^11^ As a result, employers would call upon local authorities who would impose a settlement far short of workers’ claims.

But Adam Smith went further. After having described the industrial discipline of the day, he also noted that there was a certain point below which wages could not be brought down. Workers had to earn enough to bring up a family, which might be at least twice the minimum needed for survival. What else is this than a link between organizing rights and a “living wage”?

## A History of Linkages

In the early twentieth Century throughout Europe, the demand for an eight hour day was closely associated with the call for universal suffrage. Freedom to express a political view is an inalienable part of freedom of association. Another fundamental trade union demand was the right of workers to voluntary migration while securing equal treatment, i.e. non-discrimination, with national workers.

Factory safety has an intimate link with freedom of association. It is logical that workers are engaged through health and safety committees but also ensuring that special safety representatives, elected by the workers, have enough authority to intervene in a dangerous situation. This is one area where a look at history reminds us how much work remains to be done.

In 1911, a fire in New York City killed 146 workers, mainly young immigrant women, in the Triangle Shirtwaist Factory. Locked doors and lack of safety measures contributed to the high toll. The tragedy led soon thereafter to the adoption of factory legislation in New York. But it also had another consequence: it intensified organizing activities by the Ladies International Garment Workers’ Union, a prominent American trade union. This showed that labour inspection, one of the original priorities of the ILO, is only one part of the solution. Empowering workers to defend their interests is another, and the most direct road to that is through agreements and bargaining between employers and duly authorized workers’ representatives.

Fast forward to our days: we continue to experience similar factory fires and disasters in South Asia. This culminated with the collapse in 2013 of the Rana Plaza factory building in Dhaka, Bangladesh, with a death toll of 1134. International accords for compensation and increased factory safety have since been concluded in Bangladesh,^12^ but there has been little progress in the fundamental question of not only hindering but assisting union organization. Also, as with the Triangle Shirtwaist Factory fire, factory owners have rarely if at all been made to answer for their responsibilities.

## Taking a “First-Things-First” Approach

As noted above, for the ILO the main way of diminishing the use of child labour lay in applying standards on the minimum age for employment. When the opening up of global markets made child labour a priority, this standard was seen not to address with sufficient vigour the fact that large numbers of working children were well under any established minimum age, in conditions preventing education and harmful to their health and morals.

A “first-things-first” approach^13^ was applied to legally prohibited and hazardous child labour by the Worst Forms of Child Labour Convention No. 182, which at the time of writing is only one short of ratification by all ILO member States. While the abolition of all child labour has remained the target, urgent action was to be taken against its worst forms. This time a degree of flexibility was accorded by the traditional provision that governments—together with the social partners—should determine what kind of work was hazardous and thus eliminated as a priority.

Addressing particularly urgent issues has characterized the standard setting of the ILO in the era of globalization. The ILO has increasingly focused on vulnerable groups and urgencies with such instruments as the HIV/AIDS Recommendation in 2000 and the Conventions on Domestic Workers in 2010 and Violence at Work 2019. The Protocol against trafficking, attached in 2014 to the Forced Labour Convention No. 29, belongs to this same category.

Different areas of social policy were extensively set out by Convention No. 102 of 1952, which despite wide policy agreement has gained only a limited number of ratifications. In line with the “first-things-first” approach, a normative move towards universal social security coverage was undertaken by the adoption of a Recommendation on Social Protection Floors, 2012 (No. 202). While it is not possible to globally determine minimum protection levels that could be applicable to all, the Recommendation set out the principle that everyone in every country should have at least a basic level of protection. The Recommendation does not undermine or set aside the comprehensive Social Security Convention No. 102, which remains a state of the art instrument. Nor does it aim to set minimum levels which, if enforced as the minimum everywhere, would inevitably put more pressure on those who already have achieved higher levels of protection.

At the same time the coverage of standards has extended to the informal economy with a Recommendation adopted in 2015. The Recommendation aims to increase the organizing of all workers and economic units—enterprises and households—in the entire informal economy where 62% of the global workforce is to be found. Of particular importance, too, is the Decent Work for Peace and Resilience Recommendation, 2017 (No. 205). This replaced a Recommendation adopted by the International Labour Conference in 1944 on transition from war to peace. The Recommendation outlines measures for relief and reconstruction after not only war and conflict but also natural disasters. The employment and social effects of the COVID-19 pandemic have made this Recommendation particularly topical.

In the area of maritime labour, all existing instruments—binding and nonbinding—were in 2006 combined in a coherent, living and continuously updated instrument for an inherently globalized sector of work. It incorporates both obligatory measures and recommendations and has an in-built review and revision mechanism. The large number of ratifications of this Convention is persuasive evidence of the viability of the approach, although it may not be transferrable to other less international economic sectors.

In adopting the Centenary Declaration on the Future of Work, the 2019 International Labour Conference requested the Governing Body “to consider, as soon as possible, proposals for including safe and healthy working conditions in the ILO’s framework of fundamental principles and rights at work”.^14^ This does not necessarily call for amending the 1998 Declaration in some way.

One of the purposes of the 1998 Declaration was to achieve cohesion between the four categories of rights that it covers. With occupational safety and health, the next step might well be achieving more cohesion between existing standards and policy instruments. This could be done in several ways. A state of the art overview could lead into drafting a summary of principles which are common to existing instruments and can be linked to the ILO Constitution. The result could be either a Declaration or some other authoritative document, which could include a cross-reference to the 1998 Declaration. It would also be feasible to apply the “first-things-first” approach to determine action against the most dangerous but common hazards, rather in the way that the Social Floors Recommendation has done. The next recurrent discussion on occupational safety and health in the International Labour Conference would be a good place to propose decisions on future steps.

## Where to Go Next with Standards?

The question has been often posed: to what extent has the International Labour Code already been completed? I would argue that the answer is, to some extent yes but there is always unfished business. In the last hundred years, we have covered virtually all of the basic elements of social justice as identified by the Constitution of the ILO. Yet we have to continue to adapt standards so that they maintain their relevance to structural, technological, economic and political changes in the world economy.

Work is a moving target, and the International Labour Code will never be complete in any case. Some of the early maritime labour standards covered occupations which no longer exist. Some of the early protective approaches to the work of women, such as prohibiting night work, have been replaced by demands for equality and safety. Furthermore, the necessary adaptation does not always need to be done—and in some cases cannot be done—by drafting new standards.

The potential of Recommendations is being harnessed. But this may not yet be sufficiently matched by the fact that Recommendations, as standards, are subject to overview by the standards supervisory mechanism. It would be entirely feasible to focus more on the information that can be obtained under Article 19 of the Constitution from not only Recommendations but also non-ratified Conventions.

We cannot run behind each change in labour conditions, trying to put up a new standard here or there. If we try this, by the time a Convention is adopted and effective, the issue may well have mutated into something else. What we need instead is a combination of standards and social dialogue, which is the singular contribution of the ILO over the last hundred years.

## Standards in the Twenty-First Century

In the 1970s, the ILO adopted 23 Conventions. In the next decade, the number of Conventions was 16. In the 1990s, a total of 13 Conventions were adopted, and the number of stand-alone Recommendations increased. In the first decade of this millennium six Conventions were adopted. The second decade of the millennium has seen two Conventions, one Protocol and three stand-alone Recommendations. On the other hand, since 1998 the ILO has adopted three Declarations. These are not standards in the legal sense of the word, but one of their aims is to give instructions on how standards should be universally applied.

Thirty years ago, the combination of globalizing markets and the end of Cold War confrontation brought the issue of regulation and labour standards into new focus. Ever since this soul-searching started, standard-setting has been behaving differently.

In the first 20 years of this century, all the different instruments available to the ILO have been used. The latest Conventions are on such issues as domestic workers and violence at work. Recent stand-alone Recommendations deal with HIV/AIDS in the workplace, social protection floors, the informal economy and post-conflict transition to peace. The concept of Decent Work, launched by ILO Director-General Juan Somavia and pursued by his successor Guy Ryder has entered the body of standards. Through the Protocol to the 1930 Forced Labour Convention, the ILO covered the issue of human trafficking and also revised the Convention. In addition, in 2018 the Conference for the first time abrogated obsolete Conventions.

The work on international labour standards is not drying up. It is alive and well, and is adapting to the economic, social, political and technological changes of a globalized economy.

## The State and the Social Partners

It is necessary to further reflect on the role of the state as well as the ILO’s constituents, trade unions and employers. When in 2002 President Tarja Halonen came to Geneva to co-chair for the first time the ILO’s World Commission on the Social Dimensions of Globalization, she defined the task in a simple and sensible way. The aim of the exercise should be to rehabilitate the social state. Since the technological changes of the 1980s and post-Cold War liberalization, with its shock therapies, the role of the state in guaranteeing basic welfare and services for its citizens has been under attack. Yet there are limits to how far the state can abandon or outsource its responsibilities. Crisis situations bring us rapidly to those limits.

All states of the European and Central Asian region have ratified the eight Conventions on Fundamental Principles and Rights at Work. This is a reaffirmation of the rule of law in their systems, which continue to aspire to the aims of a welfare state. Yet the economic, political and structural shocks of the last decade have had serious consequences. A feature of globalization is that prosperity and deprivation not only coexist but are increasing in all societies, whatever label we give to them.

One of the lessons of 20 years of technical cooperation on fundamental principles and rights is that their strength and sustainability depend on how they affect the individual and collective rights of workers and employers. For instance, freedom of association needs to be followed up by social dialogue and institution building. Eradicating discrimination calls for a competent labour inspectorate, gender equality and policies on migration and supporting different vulnerable groups. It also calls for a continuous effort to raise the social protection floors of societies. A case in point is the way in which health and safety measures have to rely on enabling workers to survey and intervene when conditions are inadequate and downright dangerous. This calls for competence-building for workers—and their right to consultation, negotiation and organization.

A significant proportion of labour and social issues are resolved through negotiations and social dialogue between the partners directly concerned. They make agreements which are legally binding on both parties. At times, in the discussion on what is binding and what is not, we seem to forget that voluntary bargaining produces legally binding outcomes.

International labour standards are negotiated, implemented and supervised through a process which is at times either bipartite or tripartite. With a combination of private and public actors and national and global pressures, participation by everyone is crucial. It is at workplaces where a universal principle becomes reality. Once we are clear about the principles, those directly concerned—employers and trade unions—are the ones who should find practical solutions to be applied. As before, it is in the self-interest of the state to support these processes with institutional and economic support.

Consequently, the worst thing that we can do is to saw off the branch of social dialogue. Our economic salvation and prosperity lies in the way in which rules—such as international labour standards—are implemented in practice. The state will continue to play a role in this, but we should increasingly enable the constituents of the ILO to determine how this is done. No central authority can have an overview and control of the endless amount of individual situations faced by employers, workers and local institutions. Here lies the real social responsibility of both management and labour.

## Annex: A Timeline of the ILO and Globalization

### The Founding of the ILO

The ILO was founded in 1919 in response to demands that clauses dealing with labour would be included in the Peace Treaty after the First World War. In this way the ILO was to provide the social dimension of the settlement after the Great War, which was supposed to end all wars.

The founding principles of the ILO were based on proposals made during and after the War by the trade unions of the Allied and Central Powers as well as neutral countries. They were particularly clearly expressed at conferences held in Leeds (1916) and Berne (1917 and 1919). Most of these goals were shared by Labour Parties and socially progressive intellectuals, academics and politicians of the time.

The Constitution of the ILO declared that universal peace was possible only through social justice. The basic aim of the ILO was to guarantee social justice through a minimum level of standards which would avoid competition between countries at the expense of labour conditions.

One of the founders of the ILO, the Belgian legal scholar Ernst Mahaim, explained the international dimension in the following way: “The concept of international legislation is from the outset opposed to that of absolutely unrestricted international competition.The idea is to allow *relative* freedom of competition, based on some degree of equality in costs of production; certain humanitarian requirements are to be taken out of the sphere of competition. This means that health, life and human dignity are regarded as benefits of supreme value. Humanitarian ideals are given precedence over considerations of economic profit.”

Mahaim wrote these thoughts in the seminal history of the creation of the ILO, edited by James Shotwell in 1934. Shotwell—an influential negotiator on the American team in Paris in 1919—later recalled that the name of the “International Labour Organization” was something of a misnomer. What was created was an international economic organization to deal with labour problems.

The fact that different labour conditions affect trade had been discussed since the end of the slave trade and the beginning of industrialization. Labour issues were particularly urgent at the end of the First World War because of the Russian Revolution in 1917 and strikes by workers in many European countries. Tripartite cooperation thus became an acceptable alternative to conflict and revolution. This was supported by the non-revolutionary trade union movement, which was given a voice and a vote in decisions on laws and policies.

Throughout its history, the ILO has been confronted with the choice between revolt or reform. It has promoted the method of negotiations and incremental improvements as opposed to rejection and full-scale upheaval. Looking back today—with the experience of complex social, political and economic phenomena such as globalization—it has consistently searched to maximize the benefits of change while minimizing its negative consequences.

### Albert Thomas 1920–1932

A priority of the French first Director of the ILO, Albert Thomas, was to establish the ILO as an actor in its own right in international economic policy making. He was only partly successful in this, due to reluctance on the part of governments and employers. However, he succeeded in getting the ILO to the table at important economic conferences in the 1920s, thus setting a pattern for ILO participation in world governance.

Thomas was more successful with promoting the rights of trade unions, which were often still viewed with suspicion, even hostility. Thomas supported integrating the unions in economic policy making, but he was criticized by employers, by the Soviet Union as well as by authoritarian and fascist political leaders.

The development of the new science of industrial relations further underlined the need to recognize and expand the role of trade unions. In his last report, in 1932, Albert Thomas explored the potential of both economic planning and industrial relations. However, it was difficult to defend the idea of planning because of the example of the Soviet Union, which remained a political threat and a challenge due to its stated support for revolutionary action by workers. Yet the ILO could show that, in times of crisis, the state had an important role in sustaining employment and production.

The United States stayed outside the ILO in 1919 although it had been instrumental in the peace negotiations. Yet some American businessmen were interested in engaging with it in studies on scientific management.

### Depression and the New Deal 1932–1938

Unemployment was discussed regularly by the International Labour Conference and the ILO Governing Body. This focus soon widened from employment to the organization of production and the way in which economic policies were conducted.

With the Great Depression of the 1930s, the ILO’s response was close to that of John Maynard Keynes. During the time of both Albert Thomas and his successor as Director, the British Harold Butler (1932–1938), the ILO supported going beyond unemployment insurance by public works programmes. These were a key element of Franklin D. Roosevelt’s New Deal in the United States. They were also in line with the ILO’s belief in an organized but democratic managed market economy, which helped convince the USA to join the ILO in 1934.

### Interruption by War

The main contribution of the American Director of the ILO, John Winant (1938–1941) was to evacuate the ILO from Geneva to Montreal, Canada. The ILO faced an existential threat. Germany’s ambitions included replacing the ILO by a fascist international labour organization.

The ILO was unable to achieve an in-depth conference discussion on tripartite cooperation before the Second World War interrupted its work. The experiences and lessons of war-time tripartite cooperation in Allied and neutral countries were examined at an extraordinary ILO Conference in 1941 in New York. Edward Phelan, the Irish Acting Director and later Director General (1941–1948) presented a report arguing why the ILO and tripartite cooperation needed to have a role for when the time came for reconstruction after the war.

The ILO’s philosophy was in line with the democratic principles of the Allied countries. The Atlantic Charter of Roosevelt and Churchill, drafted in 1941, contained a reference to labour standards.

The 1944 International Labour Conference in Philadelphia made the ILO fully functional again. The Philadelphia Declaration strengthened the ILO’s claim to be involved in all policy making. Tripartite cooperation was not limited to social questions; it extended to international economic policy as well as questions of war and peace. Eventually, the construction of European institutions involved consultative arrangements with employers and workers.

In this way the post-war role of the ILO was to provide the social dimension of reconstruction. In 1919–1939 tripartite cooperation had largely been a process at the international level, but now it became recognized as a national tool.

Despite the misgivings of the Soviet Union—due to the presence of free employers and independent trade unions—the ILO’s status was confirmed as the first Specialized Agency of the United Nations family in 1946.

The notion of human rights was expressed after Philadelphia in the Universal Declaration of Human Rights which the United Nations adopted in 1948. The ILO adopted Conventions on freedom of association and the right to collective bargaining, forced labour and non-discrimination (including equal pay for work of equal value) in the 1940s and 1950s. If they had been adopted by the United Nations, employers and trade unions would not have had a role in drafting, promoting and supervising them.

### The Golden Decades

The three decades following World War II are sometimes called “golden” or “glorious”. This applied above all to Europe and the rest of the OECD area. The ILO was managed by the American David Morse (1948–1970), who had first-hand experience of German reconstruction. The global situation started to change rapidly due to the wave of decolonization, which transformed the composition of the ILO. The multilateral system of the United Nations differed radically from that of the League of Nations.

The Western market economy produced unprecedented growth, including in terms of incomes and social security for workers who increasingly formed the middle class of industrialized societies. While decolonization changed the global composition of states, it did not bring prosperity and security which would have been comparable to reconstruction in Europe and Japan. It created new needs and expectations for vast numbers of people, but it also provoked high levels of frustration.

The ILO participated from the beginning in the technical cooperation activities of the United Nations, which were aimed at helping the newly independent countries gain the necessary know-how to run their economies and societies. During the first two decades of its existence, the ILO had concentrated on international labour standards and knowledge and research. Now technical cooperation meant working physically on the ground, with governments, employers and trade unions in countries which had gained political independence but needed advice and help to manage their economy and society.

The human rights aspect of decolonisation was particularly marked in the case of *apartheid* in South Africa. African nations’ opposition to *apartheid* almost blew up the 1963 International Labour Conference. It was the last vestige of colonial rule. In the 1920s and 1930s, Workers’ Group members had pressured the ILO to take a stronger stand against forced labour in the colonies. The governments’ general attitude had been to limit and regulate forced labour; the trade unions insisted successfully that the aim was to abolish it.

### Rivalries Between Groups

Sovereignty was a significant concern for many of the newly independent countries. In many of them, the new leaders had participated in national liberation movements. They wanted assistance but not patronizing while the former imperial powers did not want to give up the benefits that years of dependence had generated. At the same time, the Cold War produced rivalries between the market economies and socialist systems.

The collective answer of the developing world was to aim at a New International Economic Order (NIEO). One of its cornerstones was sovereignty and strong government, which did not tolerate much internal opposition. Consequently, the NIEO was never accepted as such in the ILO where both employers and workers insisted on their autonomy and respect for international labour standards. In the ILO, the workers wanted to speak of a new international “economic and social order”.

The workers generally agreed with the developing countries on economic policies, while on standards and rights they agreed with the industrialized democracies as well as with employers who opposed state intervention. Much of the support by employers for freedom of association in the ILO during the post-war reconstruction era was due to their insistence on free enterprise as opposed to communist state management.

### Development or Rather Lack of It

In the newly independent countries, much of the body of international labour standards applied only for part of the economy. Independence alone and the growth of now indigenous activities did not produce jobs beyond a small circle. Industrialization thus increasingly proved not to be the solution. Neither the liberal market economy model nor the socialist development model could be successfully replicated in the developing world.

When the expected outcomes did not materialize, the ILO launched a World Employment Programme in the 1960s. This was a multidisciplinary approach to economic and social development problems, with an attempt at understanding the underlying factors of development.

At the same time, powerful new economic actors emerged on the world scene. The most significant were multinational enterprises, a factor with potential economic as well as political consequences. Some of them served the interests of their home countries, to the detriment of host governments. The most notable case was Chile, where a US multinational enterprise was involved in the coup d’état against president Salvador Allende in September 1973.

As a result, a new consensus emerged: there had to be both national and international guidance for the behaviour of multinational enterprises. The basic aim was to maximize the benefits of activities by international enterprises while minimizing their negative effects. This same principle has been later applied to technological change, structural adjustment—and to globalization.

Up to the end of the 1970s, the main hypothesis for future development was that of a mixed economy, with coexistence between private and public activities. In Sweden, plans were developed for wage earners’ funds which would control much of the economy. The German practice of co-determination, *Mitbestimmung,* inspired different proposals for industrial democracy. This was not socialism; at most it was convergence.

The ILO, with capitalist, socialist and developing countries and different configurations of their workers and employers, tried to maintain a balance. The ideal outcome would have been convergence between the economic systems. But this did not happen. In fact, when capitalism and socialism “converged”, this did not take place in the centre—where it might have been expected—but on the extremes of the market economy model.

### Market Forces Reassert Themselves (1980–1989)

In the late 1970s the socially successful European economy started lagging behind. The same happened in Japan. A number of developing countries (such as Mexico) were confronted with debt crises. This led to an ideological conclusion: the industrialized countries and their economic models had allegedly developed rigidities because they had too much regulation for social and labour aims. It became fashionable again to speak of encouraging what John Maynard Keynes had critically called “the animal spirits” of the market.

This return to the markets was accompanied by significant technological change. This affected the way enterprises functioned. The model of management changed due to information and communication technology. Increased computerization enabled real time control of production in several locations in different countries. At the same time, traditional links were being cut between labour and management in the work collective.

Attempts to deal with what was labelled structural adjustment did not produce much tripartite consensus in the ILO. As Director-General (1974–1989), Francis Blanchard, from France, was successful with advancing technical cooperation with the developing world. He also brought China back into the sphere of ILO activities. However, he experienced more difficulties when the expectations and priorities of industrialized and developing countries differed increasingly from one another. At the same time, they were experiencing difficulties arising out of lack of employment and growth and persistent poverty.

The ILO was very much focused on the developing world while the basis of its activities in the industrialized countries weakened. The centralized state-led model of the communist countries was about to disappear. Since 1919 the ILO had played a significant role as an alternative to communism. Now, however, the threat of revolution seemed no longer to be there. Instead, consensus strengthened around a market economy model with more flexible social and labour rules.

In the industrialized countries, the post-World War II generation had come to believe that the ILO’s basic task was to deal with the developing world. That was where employment growth and social stability were needed. However, the same problems that they tried to cope with now started to resurface at home.

The dynamics in the 1980s in Europe were dominated on the one side by Margaret Thatcher’s United Kingdom, with a bitter miners’ strike and lingering class conflict. At the same time, the internal market of the European Economic Community (later the EU) was built up by Commission President Jacques Delors through the introduction of social dialogue. Delors believed that a deepening internal market needed the support of the social partners, especially the trade unions. Margaret Thatcher was set on resurrecting a hard market economy despite trade union resistance. The beginnings of Brexit lie in these contradictory views about how to deal with labour and social questions.

Since the early days of the ILO, it had been accepted that social and employment concerns could legitimately slow down economic activity. In the 1980s, this belief was being revisited. The factors that affected it were the increasing role of private economic interests, especially MNEs, which were usually supported by the governments of their home countries. However, the MNEs grew more transnational and less dependent on national governments. The alternatives were limited, as neither the communist model nor centralized public management produced prosperity.

By the end of the Cold War, multinational enterprises had been recognized as a new and powerful international phenomenon. Attempts by the United Nations, the ILO and the OECD to regulate these multinational forces had shown the extent to which the rules of the game could not be enforced.

After three decades of economic, social and employment growth, the 1980s led to a rehabilitation of market forces and private entrepreneurship and a demand for more flexible social and labour standards. The process of setting up the ILO in 1919 had proved that international labour standards could be implemented only through national laws. There was no applicable jurisdiction over multinational entities. As the UN, the ILO and the OECD soon had to accept, the only way to get beyond non-binding recommendations was through follow-up mechanisms which provided for consultation and dialogue.

### The Brave New Global Market Economy

The Berlin Wall started to crack at least a decade before it actually came down in November 1989. Parallel to the new technological and market-oriented policy in the West, the socialist countries had started realizing that their system no longer worked. In China, the reforms of the late 1970s led to inviting multinational enterprises to join partnerships between the market and social and political state control. The socialist countries of Europe, and Hungary in particular, sought a new balance by encouraging some market forces.

The systemic change led to both economic change and political democratization. However, the change went well beyond the dismantling of the European barriers between communism and the free market economies. Democratization took place almost everywhere. The *apartheid* system in South Africa was dismantled. Single-party systems in Francophone Africa came to an end, and former British colonies carried out multiparty elections. In Latin America there was a shift to the left, away from military rule.

On the economic side, the former socialist countries needed a radical new start. At the same time, the developing countries that entered the orbit of global trade wanted to gain maximum advantage in terms of what they had—one of them being abundant labour. The new global market economy came about through a rush to gain maximum advantage. One consequence of the changes was promotion of trade through the setting up of a new World Trade Organization in 1995. The expectations of the new participants in the global markets were vast. The general assumption was that there would be significant trade liberalization and no new trade restrictions.

Throughout the 1980s, the emphasis on the sovereignty of independent countries had run into conflict with ever clearer signs of interdependence. The phenomenon of multinational enterprises had increased awareness of the constraints created by private entities, which were increasingly outside state control. The liberalization of financial markets and capital movements increased this trend. The leading industrialized governments pursued pro-market policies which either by design or as a consequence diminished the state’s role in the economy and narrowed the space for social and labour policies.

### Search for Social Rules of Competition

In contrast to the endings of the world wars in 1919 or 1945, there was no peace treaty when the Cold War evaporated, no settlement, and no social dimension. This was not on the agenda of the United Nations or other international bodies dealing with transition from state controlled to market economies and the opening of markets. The closest the world community came to that were the negotiations for creating a real World Trade Organization. There was only limited scope for social concerns in the process that led to the WTO in 1995.

In the industrialized countries, employment and prosperity of workers were being threatened by the new and unregulated operation of the global production system. When walls came down, what appeared on the other side was that the problems that the ILO had wrestled with throughout its lifetime were still there: child labour, forced labour, discrimination and limitations on freedom of association. Not only were they there: the trade of several countries was boosted by unacceptable labour standards.

The result was a discussion on a possible social clause in trade agreements so that trade liberalization would be conditioned by respect for certain fundamental labour standards. There were different views on whether this should be managed by the WTO or the ILO, or both, and how.

Michel Hansenne, from Belgium, who was Director-General of the ILO 1989–1999, presented to the 1974 International Labour Conference a report which recognized the new context and explored ways to deal with it. He disagreed with the idea of a social clause in the WTO but maintained that the social dimension of trade liberalization could be encouraged. The immediate focus was on trade, but the agenda was soon redirected into a more general discussion on globalization. It was at this stage that the concept of globalization entered the multilateral vocabulary.

The ILO has always striven to find a negotiated, consensus-based solution to situations which are inherently conflictual. Since the early 1990s the ILO had increasingly tried to help put an end to child labour, which had become an acute issue due to the opening of world markets. There were popular calls for banning the imports of all goods produced by children or boycotts of imports from countries which did nothing against child labour.

In stressing technical cooperation to eliminate child labour, to monitor production and education and training as an alternative for children who had not yet finished their basic schooling, the ILO acted in exactly the way in which labour standards and tripartite cooperation were used as an alternative to revolutions and disruption. In 1919 there had been attempts to export (and import) revolutions. This time the question was of trade, which was seen to undermine incomes and minimum safety for workers.

The WTO stated categorically that labour standards were a matter for the ILO and called on all Member States to assist the ILO in setting and supervising them. This led to a Declaration on Fundamental Principles and Rights at Work, adopted by the International Labour Conference in June 1998. The Declaration determined that every member of the ILO had to respect the principles of freedom of association and the right to collective bargaining, abolition of forced labour, elimination of child labour and non-discrimination in employment and occupation (including equal remuneration).

The novelty of the Declaration was that it concerned not only those countries that had ratified the Conventions of these four human rights categories. The obligations extended to all. Those who had no legal obligations through ratifications had the obligation, arising out of the ILO Constitution, to strive to realize the principles. Reports were requested and examined from all countries. This led to debate, especially on China, the Gulf States and the United States. In addition, the Declaration recommended technical cooperation as the way to resolve shortcomings in the application of fundamental standards. This opened the road to using technical cooperation on a large scale for human rights problems in the labour field. In general, the countries concerned recognized that technical cooperation could be a real alternative to trade or other boycotts.

### Decent Work

The notion of Decent Work was developed by the Chilean Director-General of the ILO, Juan Somavia, in his first report upon taking office in 1999. The aim was not to change the aims of the ILO but to repackage them. The ILO budget had expanded to 39 major programmes, and Somavia concluded that an organization with so many priorities had no priorities at all. He cut the number to four: employment, social protection, standards, and social dialogue. “Decent Work” was a convenient way to express these aims—but especially after Amartya Sen’s recognition of it as a development paradigm at the 1999 International Labour Conference, it became the framework for managing and carrying out ILO activities.

Decent Work remains a shorthand way of expressing the ILO’s purpose. It also underlines the interdependence between actions in these fields. To succeed in the sustainable promotion of Decent Work, it was necessary to engage all four areas of action. Gradually the notion of Decent Work became accepted by the multilateral system, including the United Nations.

### The Globalization Commission 2002–2004

The international dimension of Decent Work—or in other words, the link between decent work and globalization—was examined in depth by a tripartite World Commission on the Social Dimension of Globalization, co-chaired by Presidents Tarja Halonen of Finland and Benjamin Mkapa of Tanzania. The Commission surveyed the full range of social and labour issues. It built on the traditional aim of maximizing benefits and minimizing negative effects. One of its messages was that, to be acceptable, globalization must be fair—and it must be seen to be fair.

The World Commission also commented on the fundamental principles and rights at work of the 1998 Declaration, pushing them one step further. While the Declaration had noted that these rights should not be used for protectionist purposes, the Commission added that nor should denial of these rights be used to gain competitive advantage.

In 2008, this principle was included in the ILO Declaration on Social Justice for a Fair Globalization. This Declaration was designed to be the compass for promotion of fair globalization based on Decent Work.

### The Financial Crisis and the Global Jobs Pact

The 2008 Declaration underlines that the four objectives of the ILO—the objectives of Decent Work—are inseparable, interrelated and mutually supportive. This probably was the strongest recognition of interdependence up to that date in the official positions of the ILO. Furthermore, it created a follow-up in the International Labour Conference. Henceforth each strategic objective would be regularly and in turn discussed by the Conference. The aim was to determine how the issues have developed, how ILO programmes were working, and what improvements—including new or revised international labour standards—would be needed.

So far this system of recurrent items has led to adoption of a Recommendation on Social Protection Floors; a protocol against Trafficking of people for the Forced Labour Convention 1930 (No. 29); and an agreement to work further on labour conditions in global supply chains. In other words, the different negative aspects of globalization are being tackled by the ILO with concrete normative action and technical cooperation.

The era of globalization provoked tripartite discussions which, in turn, led to instruments and action on which there was broad consensus. A consensus was reached on the Global Jobs Pact, adopted at the International Labour Conference in 2009, which included a Jobs Summit of several Presidents. However, it did not lead to extensive follow-up measures. This is all the more regrettable, as the initiative came from employers—especially from national employers who had started feeling the pressures of the financial crisis. Yet one consequence has been that, since 2009, the ILO has regularly been invited to participate in the G20 Leaders’ Summit meetings. The Labour and Social Affairs Ministers of the G20 group also meet, and they consult with international employers’ organizations and trade unions.

There is a difference in the approach of the Employers’ and Workers’ Groups. For the workers, the natural model of a follow-up has been the international labour standards system, which can reach out to legal or at least semi-legal conclusions concerning violations of rights. The employers’ vision generally is more of systems of information and consultation, without sanctions or legal obligations. It is not surprising that, for a hundred years, the workers have been advocating adoption of Conventions whereas, for the employers, the preferred form of instrument has been a Recommendation.

### The Future of Work and the Centenary Declaration

Juan Somavia’s successor, Guy Ryder from Great Britain, was elected Director-General of the ILO in 2012. He has directed much attention to the future of work. A new tripartite Global Commission—set up to study the topic—made its recommendations in January 2019. They were the basis for the Centenary Declaration, adopted by the International Labour Conference in June 2019.

In that Declaration, the word “globalization” is mentioned only twice. Does this mean that priorities have shifted? The context is more important than the frequency of mention. The Declaration is quite honest in asserting the basis of the world economy as it has developed over the last three decades. It supports the private sector as “a principal source of economic growth and job creation” while the public sector is “a significant employer and provider of quality public services”.

This could be seen as the definite end to a period of competition between private and public development models. In this context, Decent Work appears as the key to sustainable development, conflict resolution and prevention, and cohesive nation building. In the context of globalization, the Centenary Declaration reaches out to one of the main Constitutional assertions, namely that the failure of any country to treat its workers humanely is an obstacle to all other countries which desire to do so. Fittingly, the Declaration weaves together the threads from over one hundred years to produce a narrative which, while not new, is the result of continuous review and updating.

In this light, it is logical that after the years of adjusting the principles of social justice to the challenges of a globalized world, it is important to determine what kind of concrete action needs to be taken. The Conference of 2019 adopted a new Convention on Violence and Harassment at Work. This Convention (No. 190) reminds us that freedom from violence and harassment at work is a human right, and in working life the rules have to cover everyone, irrespective of the contractual form of their employment. In addition, the Conference asked for further strengthening of the right to workplaces with guaranteed proper conditions of occupational safety and health.

This can be seen as a logical trajectory for the ILO. A hundred years ago the ILO was first preoccupied by such questions as hours of work, minimum age for employment, and protection against unemployment as well as conditions before and after active years of work. The brutality of World War II highlighted the role of international labour standards for democracy, thus promoting recognition of universal human rights. This was followed by decolonization and technical assistance and, later on, the search for social principles of globalization.

The principles have been clarified but have not changed. Their application must be adapted to economic and social realities—both national and international. The ILO would be well advised to move ahead with as concrete application as possible of the principles which were for the first time adopted in its Constitution in 1919 and recognized as guidelines for social justice ever since. The rule of law must be maintained as the basic framework. Within it, any consensus at the parliamentary and macro-economic levels needs to be translated into specific agreements and real action at the national, sectoral and local level. Tripartite cooperation and collective bargaining play a crucial role here. This is the way to promote universal principles at the level of workplaces and enterprises—and society in general.

## Footnotes

1 ILO, Declaration on Fundamental Principles and Rights at Work. Adopted by the International Labour Conference, 86th Session, Geneva, 18 June 1998 (Annex revised 15 June 2010).

2 Tapiola (2018).

3 Article 427, Section II, Chapter VI, Part XIII of the Treaty of Peace of Versailles 1919.

4 Ibid.

5 ILO, Resolution concerning the Protection of Trade Union Rights. Adopted by the International Labour Conference, 38th Session, Geneva, 1–23 June 1955.

6 Morse (1969), pp. 57–59.

7 OECD (1996).

8 UN (1995), para 54 (b).

9 Office of the United States Trade Representative (2019).

10 Smith (2012), p. 71.

11 Ibid., p. 72.

12 Accord on Fire and Building Safety in Bangladesh, 2013. Available at: https://admin.bangladeshaccord.org/wp-content/uploads/2018/08/2013-Accord.pdf. Accessed 4 May 2020.

13 ILO (1996).

14 ILO, Resolution on the ILO Centenary Declaration for the Future of Work. International Labour Conference, 108th Session, Geneva, 21 June 2019.
